# A Multi-Site Study of Norovirus Molecular Epidemiology in Australia and New Zealand, 2013-2014

**DOI:** 10.1371/journal.pone.0145254

**Published:** 2016-04-26

**Authors:** Kun Lee Lim, Joanne Hewitt, Alefiya Sitabkhan, John-Sebastian Eden, Jennifer Lun, Avram Levy, Juan Merif, David Smith, William D. Rawlinson, Peter A. White

**Affiliations:** 1 School of Biotechnology and Biomolecular Sciences, Faculty of Science, University of New South Wales, Sydney, Australia; 2 Molecular Laboratory, Department of Pathology, Singapore General Hospital, Singapore; 3 Institute of Environmental Science and Research, Kenepuru Science Centre, Porirua, New Zealand; 4 Marie Bashir Institute for Infectious Diseases and Biosecurity, School of Biological Sciences and Sydney Medical School, University of Sydney, Sydney, Australia; 5 PathWest Laboratory Medicine WA, Queen Elizabeth II Medical Centre, Perth, Australia; 6 School of Pathology and Laboratory Medicine, University of Western Australia, Nedlands, Australia; 7 Virology Division, SESIAHS, Department of Microbiology, Prince of Wales Hospital, Sydney, Australia; 8 School of Medical Sciences, Faculty of Medicine, University of New South Wales, Sydney, Australia; University of Hong Kong, HONG KONG

## Abstract

**Background:**

Norovirus (NoV) is the major cause of acute gastroenteritis across all age groups. In particular, variants of genogroup II, genotype 4 (GII.4) have been associated with epidemics globally, occurring approximately every three years. The pandemic GII.4 variant, Sydney 2012, was first reported in early 2012 and soon became the predominant circulating NoV strain globally. Despite its broad impact, both clinically and economically, our understanding of the fundamental diversity and mechanisms by which new NoV strains emerge remains limited. In this study, we describe the molecular epidemiological trends of NoV-associated acute gastroenteritis in Australia and New Zealand between January 2013 and June 2014.

**Methodology:**

Overall, 647 NoV-positive clinical faecal samples from 409 outbreaks and 238 unlinked cases of acute gastroenteritis were examined by RT-PCR and sequencing. Phylogenetic analysis was then performed to identify NoV capsid genotypes and to establish the temporal dominance of circulating pandemic GII.4 variants. Recombinant viruses were also identified based on analysis of the ORF1/2 overlapping region.

**Findings:**

Peaks in NoV activity were observed, however the timing of these epidemics varied between different regions. Overall, GII.4 NoVs were the dominant cause of both outbreaks and cases of NoV-associated acute gastroenteritis (63.1%, n = 408/647), with Sydney 2012 being the most common GII.4 variant identified (98.8%, n = 403/408). Of the 409 reported NoV outbreaks, aged-care facilities were the most common setting in both Western Australia (87%, n = 20/23) and New Zealand (58.1%, n = 200/344) while most of the NoV outbreaks were reported from hospitals (38%, n = 16/42) in New South Wales, Australia. An analysis of a subset of non-GII.4 viruses from all locations (125/239) showed the majority (56.8%, n = 71/125) were inter-genotype recombinants. These recombinants were surprisingly diverse and could be classified into 18 distinct recombinant types, with GII.P16/GII.13 (24% of recombinants) the most common.

**Conclusion:**

This study revealed that following its emergence in 2012, GII.4 Sydney 2012 variant continued to be the predominant cause of NoV-associated acute gastroenteritis in Australia and New Zealand between 2013 and 2014.

## Introduction

Norovirus (NoV) is the leading cause of human viral gastroenteritis globally and responsible for more than half of the gastroenteritis outbreaks that occur annually [1]. As a consequence of its high morbidity in developing countries, NoV infection is considered an important public health issue with a substantial socioeconomic burden [2–4]. In developing countries NoV is estimated to kill over 200,000 people annually; mainly children under 5 years old [5]. NoVs infect all age groups, with clinical symptoms commonly characterised by diarrhoea, projectile vomiting, fever and abdominal cramps [2, 6]. Due to its low infectious dose and environmental stability, NoVs are easily transmitted [7, 8]. Person-to-person transmission typically occurs through the faecal-oral-route and vomitus spread, hence NoV is commonly identified as the cause of outbreaks in semi-enclosed institutions such as nursing homes, schools, hospitals and cruise ships [9–12].

NoV belongs to the family *Caliciviridae* and the genus *Norovirus* which is classified into six genogroups (GI-GVI) according to phylogenetic clustering of the capsid gene [13]. An additional genogroup (GVII) that infects dogs was recently proposed [14]. Only GI, GII and GIV are known to infect humans with NoV GII strains predominant in molecular epidemiological studies [15]. Within each genogroup, NoV strains can be further classified into genotypes, with more than 36 genotypes infecting humans currently described [13].

Genogroup II, genotype 4 (GII.4) is of particular importance as it is the only genotype associated with pandemics of disease since the mid 1990’s [16]. The emergence and global spread of novel GII.4 variants are responsible for each of the six global epidemics that have occurred over the last two decades including; US 1995/96 in the late 1990s [17, 18], Farmington Hills virus in 2002 [12], Hunter virus in 2004 [19], Den Haag 2006b virus in late 2007 [20, 21], New Orleans virus in 2009 [22] and the current predominant GII.4 strain in circulation, Sydney 2012 [23, 24]. The pattern of emergence for Sydney 2012 was typical of those previous epidemic GII.4 variants [25]. Following its initial identification in Australia in March 2012, the new GII.4 variant began to displace the predecessor GII.4, New Orleans 2009, such that by late 2012, Sydney 2012 was the predominant strain in circulation globally [25]. Furthermore, the emergence of novel GII.4 viruses are associated with increases in worldwide NoV activity, as was the case with Sydney 2012 [24].

In addition to the pandemic GII.4 variants, several GII.4 variants have been identified that are associated with sporadic infections and epidemics localised to specific geographical regions. These NoV GII.4 variants include Japan 2001, Henry 2001, Asia 2003, Yerseke 2006a, Osaka 2007 and Apeldoorn 2008 [16, 26–29].

The GII.4 variants have consistently demonstrated a higher epidemiological fitness compared to other genotypes (reviewed in [30]), with a single GII.4 NoV variant predominant for a period of 2 to 3 years, through an epochal style of evolution [31]. The successful dominance of GII.4 variants has been attributed to various factors [30], including higher rates of evolution [21] and recombination [32]. Only through continuous surveillance can we gain a better understanding of the evolutionary processes shaping the emergence and spread of NoV disease, which is essential for the development and implementation of effective strategies for their control and eradication. Indeed, with a number of vaccine candidates in phase II clinical trials, it is increasingly important to characterise the NoV strains in circulation to inform the composition and assess the effectiveness of potential vaccines. Therefore, in this study we examined the molecular epidemiological trends of NoV through identification of circulating genogroups, genotypes and recombinant strains of NoV in two Australian states and throughout New Zealand, between 2013 and 2014.

## Materials and Methods

### Sample collection and identification of NoV-associated outbreaks

This study was performed under the ethical approval of the UNSW Human Research Ethics Committee (reference number UNSW HC12221). All clinical stool samples used in this study were collected as part of routine diagnostics services or surveillance programs between January 2013 and June 2014. Furthermore, patient consent was not required as specimens were de-identified and data were analysed anonymously. In Australia, samples were collected from two states, New South Wales (NSW) and Western Australia (WA). A total of 303 known positive NoV-infected stools were collected through the South Eastern Area Laboratory Services (SEALS) at the Prince of Wales Hospital in NSW (n = 159), and through the PathWest Laboratory Medicine, at the Queen Elizabeth II Medical Centre, Perth, WA (n = 144). In New Zealand, stool samples associated with gastroenteritis outbreaks were referred by New Zealand public health units to the Norovirus Reference Laboratory, Institute of Environmental Science and Research for analysis as part of New Zealand Ministry of Health national NoV outbreak surveillance. Of these, NoV-positive stool samples (n = 344) were then sequenced for genotyping and phylogenetic analysis.

In this study, an outbreak was defined as two or more NoV-positive specimens linked by time and location or institution where episodes of acute gastroenteritis occurred. Conventionally, NoV cases identified from hospital emergency wards were classified as unlinked cases, unless there was additional epidemiological information provided. A representative sample from each outbreak (n = 409) was included for molecular epidemiological studies in this investigation. Samples from individual unlinked cases of acute gastroenteritis (n = 238) in NSW and WA were also randomly selected for NoV surveillance.

### Sample processing and detection of NoV RNA

For all stool samples collected in Australia, viral RNA was extracted and a reverse transcription—polymerase chain reaction (RT-PCR) targeting the 5’ end of the capsid gene (region C) was performed, as described previously [25]. Following RT-PCR amplification, the partial capsid genes were sequenced and used for viral capsid genotyping [25]. To identify any possible antigenic variation in circulating GII.4 strains, the complete capsid gene sequences were also obtained from 48 representative GII.4 Sydney 2014 strains collected in NSW Australia. These samples were selected from the early (n = 20), middle (n = 19) and end (n = 9) of the study period using a sequencing approach described previously [33]. In order to characterise potential recombinant NoV GII strains in Australia, a nested RT-PCR was performed which amplified a 575-bp region spanning the ORF1-ORF2 overlap (nt position 4792–5366 with reference to Lordsdale virus, GenBank accession number X86557), as described previously [34]. The first round primers used were Hep170 and NV2oR [19] and then second round primers were Hep172 [19] and G2SKR [35]. Putative recombinant sequences were then compared to known reference sequences and analysed for evidence of recombination using phylogenetic approaches [21].

For the New Zealand samples, viral RNA was extracted from a clarified stool suspension (20% vol/vol) and assayed for the presence of NoV GI and GII RNA using a duplex real-time RT-PCR [36]. For genotyping at least one representative sample per outbreak was assayed using a conventional RT-PCR assay that targeted the partial polymerase gene (region B) and the N-terminus of the capsid (region C). Amplicons were then purified and sequenced, as described previously [36].

### DNA sequencing and phylogenetic analysis

For genotyping, RT-PCR amplicons were first purified using ExoSAP-IT (Affymetrix, Santa Clara, CA). Samples were directly sequenced using the BigDye-Terminator cycling methodology on an ABI 3730 DNA Analyser and a 3130XL DNA Analyser (Applied Biosystems, Carlsbad, CA) in Australia and New Zealand, respectively. Multiple alignments and phylogenetic analysis to determine the NoV genotype were performed using BioNumerics^™^, version 6.6 (Applied Maths, Kortrijk, Belgium) and MEGA version 5.2 [37]. Genotyping results were further confirmed using the automated genotyping tool from NoroNet (http://www.rivm.nl/mpf/norovirus/typingtool).

### Nucleotide sequence accession numbers

The GenBank accession number for viruses sequenced in this study are as follows: KT150976 –KT151070 and KT239551 –KT239649.

## Results

### Acute gastroenteritis outbreaks and NoV surveillance

**Fig 1:**
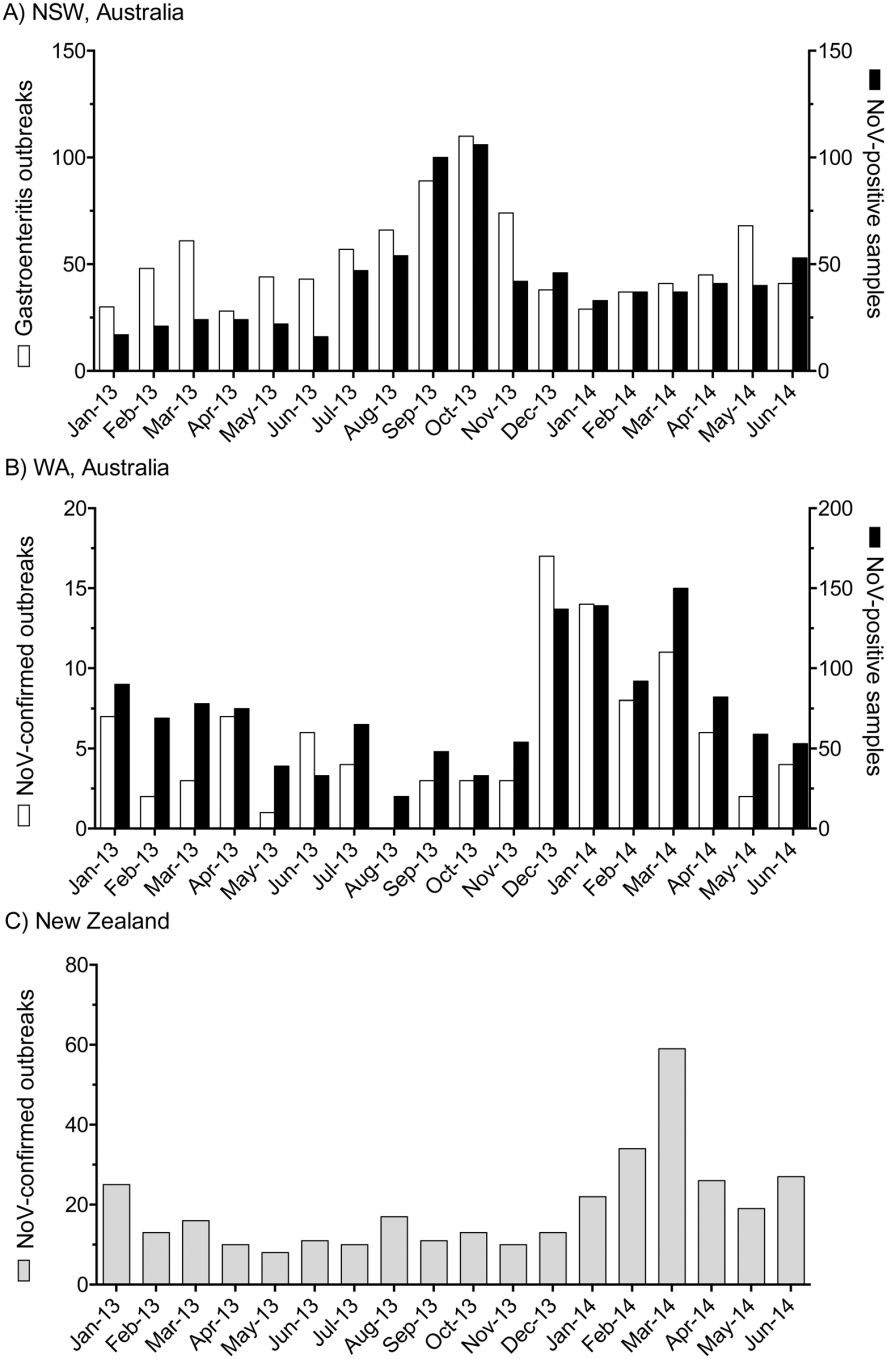
The incidence of NoV-associated gastroenteritis in NSW and WA, Australia and New Zealand from January 2013 to June 2014. (A) The number of monthly institutional gastroenteritis outbreaks reported to NSW Health (white) was compared to the monthly number of NoV-positive cases (black) reported to SEALS diagnostics laboratory Prince of Wales Hospital, NSW, Australia. (B) The number of confirmed NoV-outbreaks was compared by month to the number of NoV positive cases reported to PathWest Laboratory Medicine at the Queen Elizabeth II Medical Centre, Perth, WA, Australia. Note that the NoV-positive cases reported by both diagnostics laboratories included all individual outbreak cases and sporadic unlinked cases. (C) The number of laboratory confirmed NoV-outbreaks per month reported to the Norovirus Reference Laboratory, ESR, New Zealand from January 2013 to June 2014.

In Australia and New Zealand, NoV infection is not a notifiable disease, consequently, the incidence of NoV-associated gastroenteritis outbreaks is often underestimated. However, NoV infections are known to be the primary cause of institutional gastroenteritis outbreaks [21, 38, 39], which are required by law to be reported to public health authorities, both in Australia and New Zealand. Thus, institutional gastroenteritis outbreaks may be considered representative of the incidence of NoV infection at any one time. By assessing the number of institutional outbreaks each month and supplemented by the monthly laboratory-confirmed NoV positive cases, we investigated the NoV activity in Australia and New Zealand between January 2013 and June 2014 (Fig 1).

In NSW, Australia, institutional gastroenteritis outbreaks reported to NSW Health (n = 949) were compared monthly to laboratory confirmed NoV cases, identified by SEALS, Prince of Wales Hospital (n = 760) between January 2013 and June 2014 (Fig 1A). A large increase in gastroenteritis outbreaks was observed during the spring with a peak observed in October 2013. This increase in institutional gastroenteritis outbreaks also coincided with an increase in the number of laboratory diagnosed positive NoV cases reported for October in the same year.

In WA, Australia, confirmed NoV-associated acute gastroenteritis outbreaks (n = 101, Fig 1B) were compared by month to laboratory confirmed NoV cases identified by PathWest Laboratory Medicine at the Queen Elizabeth II Medical Centre, Perth, WA (n = 1316), between January 2013 and June 2014 (Fig 1B). Although, the peak was less defined, an increase in NoV activity was observed around the summer period, between December 2013 and March 2014 (Fig 1B). The highest number of NoV outbreaks (n = 17) reported in December 2013 while the highest number of positive NoV cases (n = 150) recorded in March 2014.

In New Zealand, a total of 344 NoV outbreaks were identified by the Norovirus Reference Laboratory, Porirua, New Zealand during the study period. Here, the monthly number of NoV outbreaks was lower during 2013 compared to 2014 with a mean of 13 outbreaks per month compared to 31 outbreaks per month in 2014 (Fig 1C). A steady increase of NoV outbreaks was seen during New Zealand summer-autumn period of 2014, with a large peak of NoV activity recorded in March 2014 (n = 59 outbreaks) (Fig 1C).

### Molecular epidemiology of NoV-associated acute gastroenteritis

**Fig 2:**
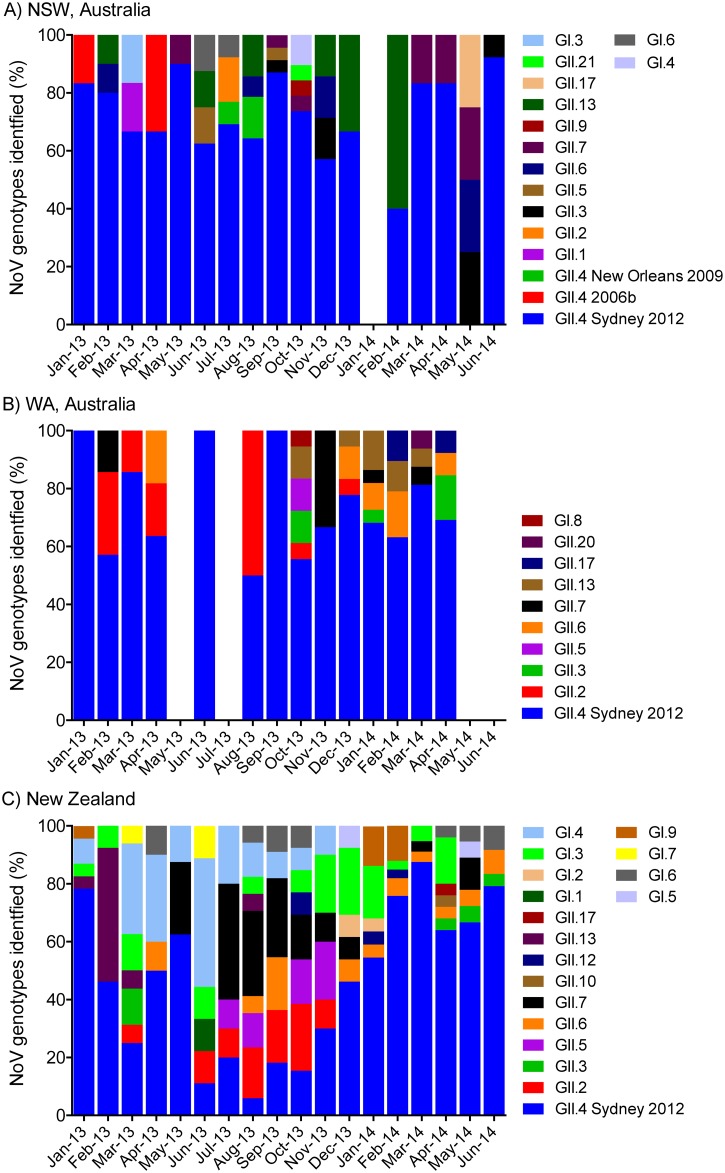
Monthly proportion of NoV genotypes identified in Australia and New Zealand between January 2013 and June 2014. The prevalence of NoV capsid genotypes and specific GII.4 variants within the 634 NoV-positive samples were compared for NSW and WA, Australia (panel A and B, respectively) and New Zealand (panel C). Samples with unknown GI or GII capsid genotypes as well as mixed GI and GII infection are excluded in this analysis (n = 13). The percentage of each NoV capsid genotype (Y-axis) is plotted by month (X-axis). Different genotypes and GII.4 variants are labelled according to the legends provided and plotted from the top down with increasing prevalence so the most predominant genotype is at the base of the graph.

A total of 647 clinical stool samples were collected from acute gastroenteritis cases in the two Australian states (NSW and WA) and New Zealand. All samples were confirmed as positive using NoV RT-PCR (Table 1). To study the predominance of NoV genotypes over time and the variation in prevalence of NoV genotypes at different geographical locations, the 5’ end of the capsid region of all 647 positive NoV samples was sequenced and genotyped (Table 1). The genotyping results were plotted by month, separately for the Australian states (Fig 2A and 2B, for NSW and WA, respectively) and New Zealand (Fig 2C). Overall, GII viruses were by far the most prevalent genogroup identified, which accounted for 87.2% (n = 564/647) of all NoV infections (Table 1).

**Table 1:** Summary of NoV genotypes identified in Australia and New Zealand during 2013–2014.

		Confirmed outbreaks		Acute gastroenteritis cases	
Location	Period of sample collection	Capsid genotype	No. of strains (%)	Capsid genotype	No. of strains (%)
New South Wales, Australia	Jan 2013–Jun 2014	GII.4	35 (83.3)	GII.4	84 (71.8)
		GII.5	2 (4.8)	GII.13	9 (7.7)
		GII.6	2 (4.8)	GII.3	6 (5.1)
		GII.1	1 (2.4)	GII.7	6 (5.1)
		GII.13	1 (2.4)	GI.3	4 (3.4)
		GI.6	1 (2.4)	GII.2	2 (1.7)
				GII.6	2 (1.7)
				GII.9	1 (0.9)
				GII.17	1 (0.9)
				GII.21	1 (0.9)
				GI.6	1 (0.9)
		***Total*:**	**42 (100)**		**117 (100)**
Western Australia, Australia	Jan 2013–Apr 2014	GII.4	22 (95.7)	GII.4	79 (65.3)
		GI.8	1 (4.3)	GII.6	10 (8.3)
				GII.13	9 (7.4)
				GII.2	8 (6.6)
				GII.3	5 (4.1)
				GII.7	4 (3.3)
				GII.17	3 (2.5)
				GII.5	2 (1.7)
				GII.20	1 (0.8)
		***Total*:**	**23 (100)**		**121 (100)**
New Zealand	Jan 2013–Jun 2014	GII.4	188 (54.8)	-	-
		GI.3	24 (7.0)		
		GII.7	22 (6.4)		
		GI.4	22 (6.4)		
		GII.6	14 (4.1)		
		GII.2	12 (3.5)		
		GII.13	9 (2.6)		
		GI.6	8 (2.3)		
		GI.9	8 (2.3)		
		GII.5	7 (2.0)		
		GII.3	5 (1.4)		
		GII.12	3 (0.9)		
		GI.2	2 (0.6)		
		GI.5	2 (0.6)		
		GI.7	2 (0.6)		
		GII.10	1 (0.3)		
		GII.17	1 (0.3)		
		GI.1	1 (0.3)		
		Mixed (GI and GII)	6 (1.7)		
		Unknown GII	6 (1.7)		
		Unknown GI	1 (0.3)		
		***Total*:**	**344 (100)**		**-**

**Fig 3:**
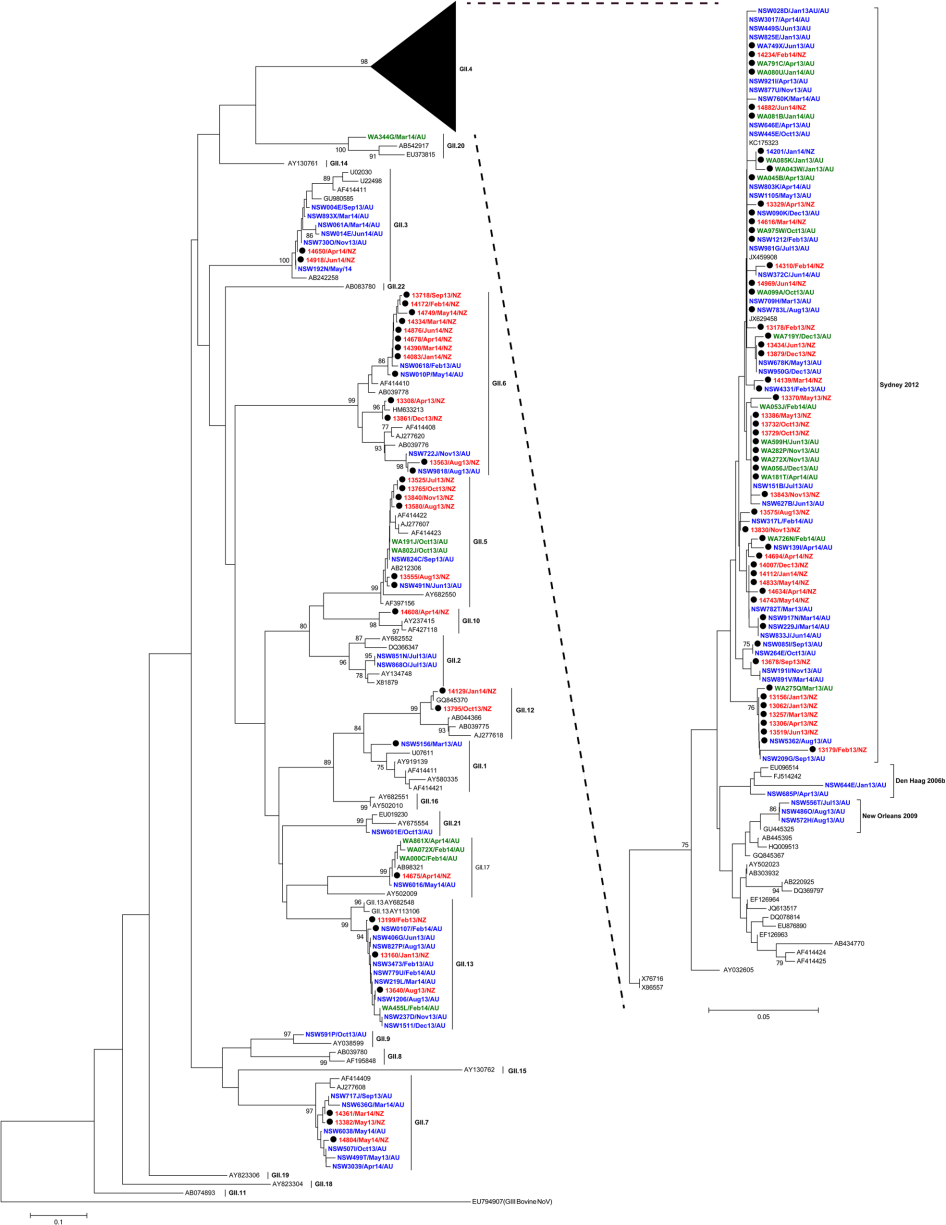
Phylogenetic analysis of NoV GII partial capsid nucleotide (nt) sequences from Australia and New Zealand. Neighbour-Joining phylogeny of sequences of the 5’ end of ORF2 was generated. NoV GII sequences (266-bp, nt position 5101–5366 with reference to Lordsdale virus, GenBank accession number X86557) are shown. Global NoV reference sequences (n = 78) genotype were obtained from GenBank and are labelled with the GenBank accession number in black. The detailed reference strain information and collection year are tabulated in S1 Table. Representative NoV sequences used for the phylogenetic analysis (n = 156) are labelled with red (New Zealand), blue (NSW Australia) and green (WA Australia), in the format of: Sample ID/Collection month and year/Country. Each NoV-associated outbreak is indicated with a black solid circle. The phylogeny was generated using programs within MEGA 5, with bootstrap values of ≥75 indicated as a percentage of 1000 replicates. The distance scale represents the number of nucleotide substitutions per site. AU—Australia; NZ—New Zealand.

**Fig 4:**
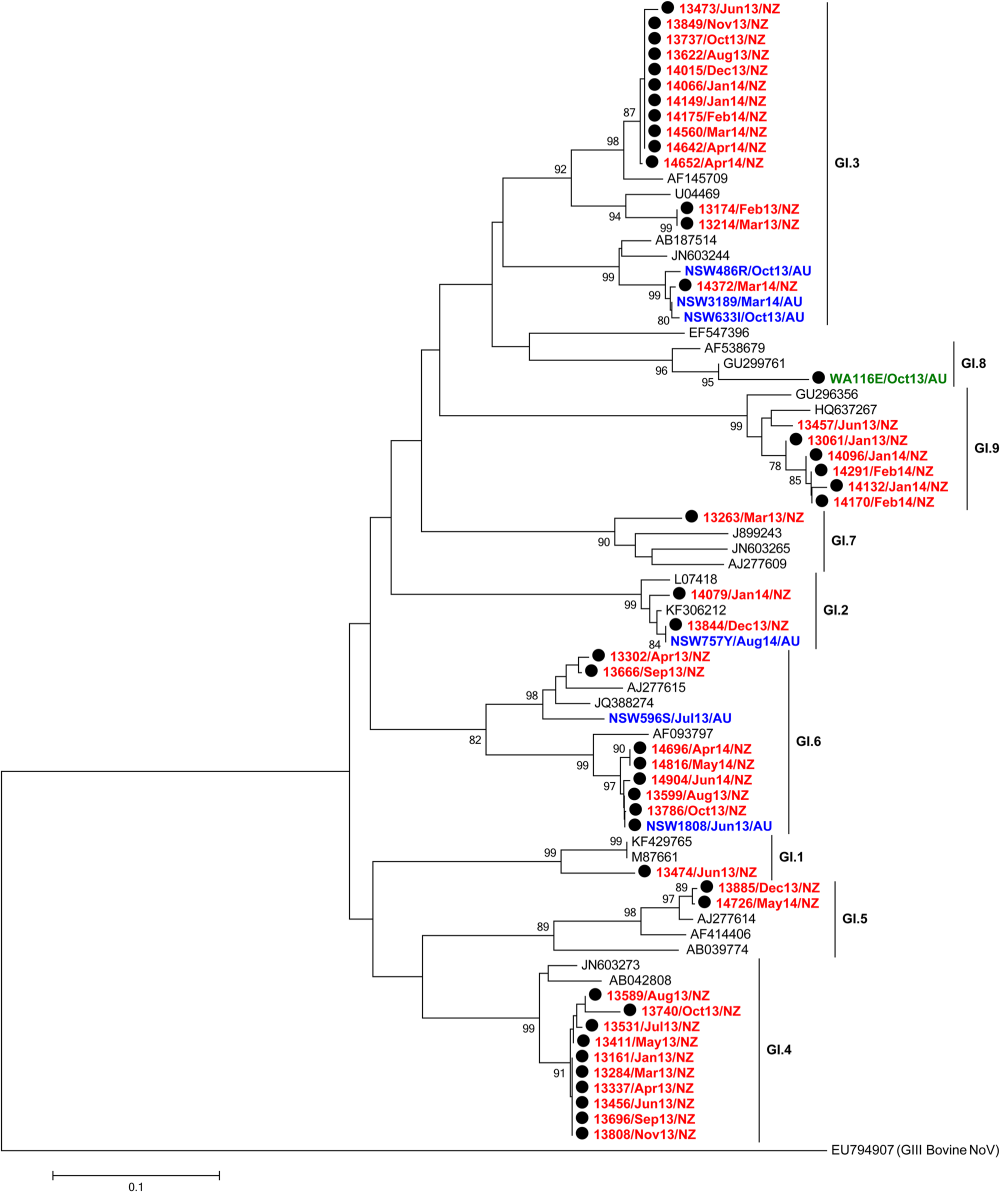
Phylogenetic analysis of NoV GI partial capsid nucleotide (nt) sequences. GI sequences (294 nt, nt position 5359–5652 with reference to Norwalk virus, GenBank accession number X87661) are shown in the neighbour-joining phylogeny of the 5’ end of ORF2. Global NoV reference sequences (n = 25) from GenBank are labelled in black while representative NoV sequences of this study (n = 50) are labelled in red (New Zealand), blue (NSW Australia) or green (WA Australia). Each NoV-associated outbreak is indicated with a black solid circle. The phylogeny was generated using programs within MEGA 5, with bootstrap values of ≥75 indicated as a percentage of 1000 replicates.

To further confirm circulating genotypes, phylogenetic trees were made using a subset of 206 samples (GI = 50 and GII = 156), selected from both outbreaks and acute gastroenteritis cases, that were representative of the distribution of NoVs identified in this study (Figs 3 and 4). This analysis included at least one sample from every individual genotype identified in every location by month. Not unexpectedly, NoV GII.4 was the most commonly identified genotype (63.1%, n = 408/647) (Figs 2 and 3), with the GII.4 Sydney 2012 variant being the major cause of both outbreaks and acute gastroenteritis cases (98.8% of GII.4 viruses detected) throughout the study period. Indeed, Sydney 2012 was the only GII.4 variant detected in WA and New Zealand (Table 1 and Fig 2). In NSW, both older GII.4 variants, Den Haag 2006b and New Orleans 2009, were also detected during the study period; however they were much less common than Sydney 2012 (Table 1, Figs 2A and 3). Den Haag 2006b and New Orleans 2009 variants were identified from January to April 2013 (2 cases) and from July to August 2013 (3 cases), respectively.

Overall, NoV GII.7 was the second most common GII capsid genotype (4.9%, n = 32/647) followed by GII.6 and GII.13 (4.3%, n = 28/647 each). Other GII capsid genotypes found in this study included; GII.2 (3.4%, n = 22/647), GII.3 (2.4%, n = 16/647), GII.5 (1.7%, n = 11/647), GII.17 (0.8%, n = 5/647), GII.1, GII.9, GII.10, GII.20 and GII.21 (0.2%, n = 1/647 each) (Figs 2 and 3). Six samples (0.9% of samples) were identified as NoV GII by the genogroup specific RT-PCR, but their exact genotypes were unable to be determined following unsuccessful attempts at sequencing.

There was important variation in prevalence of non-GII.4 strains across the different locations included in this study (Table 1). For example, in NSW, GII.13 (6.3%) was the most prevalent non-GII.4 capsid genotype followed by GII.3 and GII.7 (3.8% each). In WA, the most common non-GII.4 capsid genotype identified was GII.6 (6.9%) followed by GII.13 and GII.2, which accounted for 6.3% and 5.4% of NoV infections, respectively. Interestingly in New Zealand, the most prevalent non-GII.4 capsid genotype was not a GII virus, but a GI.3 virus (7%), followed by GII.7 and GI.4 (6.4% each). Together this highlights how the prevalence of non-GII.4 NoVs may vary across various geographical locations.

### Outbreak settings

During the study period, 409 NoV-associated outbreaks of acute gastroenteritis were identified including 65 from Australia (NSW and WA) and 344 from New Zealand. The majority of reported outbreaks occurred in aged-care facilities in both WA (87%) and New Zealand (58.1%) (Table 2). In NSW Australia, the most common outbreak settings were hospitals and followed by aged-care facilities, they accounted for 38% and 29% of the reported outbreaks in the state, respectively. Other settings involved in NoV outbreaks were residential (12%), social events, catered/commercial food settings, hostels and cruise ships (5% each), while one outbreak had an unknown setting. In WA, the second most common outbreak setting was hospitals (2 outbreaks) and followed by social event (1 outbreak). In terms of outbreaks, when considering the impact of individual NoV genotypes, GII.4 Sydney 2012 was the major cause of outbreaks in both states of Australia; associated with 89.2% (n = 58/65) of outbreaks. In New Zealand, commercial food settings (12.5%) were the second most common outbreak settings (after aged-care facilities), followed by childcare centres (11.3%). Other outbreak settings reported were hospitals (5.5%), hostel/boarding house/hotel (3.8%), residential homes (2.6%) and schools or colleges (0.3%), with 5.8% of the outbreaks settings in New Zealand unknown (Table 2). Of the 344 outbreaks reported in New Zealand, 1.7% (n = 6) were identified as mixed infection of both GI and GII genogroups. These outbreaks were from various settings including; aged-care facilities, a commercial food operator, a child-care centre, hostel and a school.

**Table 2:** NoV outbreak settings in Australia and New Zealand between January 2013 and June 2014.

Location	Outbreak settings	Number of outbreaks (%)
New South Wales, Australia	Hospital	16 (38)
	Aged-care facility	12 (29)
	Residential	5 (12)
	Commercial food operator	2 (5)
	Hostel/boarding house	2 (5)
	Cruise ship	2 (5)
	Social events	2 (5)
	Unknown	1 (2)
	***Total*:**	**42 (100)**
Western Australia, Australia	Aged-care facility	20 (87)
	Hospital	2 (9)
	Social event	1 (4)
	***Total*:**	**23 (100)**
New Zealand	Aged-care facility	200 (58.1)
	Commercial food operator	43 (12.5)
	Childcare centre	39 (11.3)
	Hospital	19 (5.5)
	Hostel/boarding house/Hotel	13 (3.8)
	Residential	9 (2.6)
	School/college	1 (0.3)
	Unknown	20 (5.8)
	***Total*:**	**344 (100)**

### Analysis of GII.4 amino acid mutations in VP1

Antigenic variation is important for the persistence of GII.4 lineages from season to season [40, 41]. We therefore examined variation in the amino acid sequence of the VP1 capsid, with a particular focus on changes in the hypervariable P2 domain that contains the major antigenic and receptor binding sites. Changes of amino acids, with R297H and D372N (epitope A), M333V (epitope B) and S393G (epitope D) were observed in S1 Fig. Overall little antigenic variation was observed and no variation at amino acid positions 294 and 368 (both in epitope A), which were previously shown to be important in the emergence of GII.4 Sydney 2012 [25, 41].

### Identification of circulating recombinant NoVs

Over the last decade an increase in the number of NoV recombinants has been reported in molecular epidemiological studies [23, 30, 42–44]. Therefore, this study specifically investigated a subset of 125 non-GII.4 samples (44 GI NoVs and 81 GII NoVs) for evidence of inter-genotype recombination by sequencing the region that spanned the ORF1/2 overlap; a known hotspot for recombination [42, 43]. Through phylogenetic analysis and separate genotyping of the partial polymerase (ORF1) and capsid (ORF2) regions, 18 inter-genotypic recombinant types were identified from the majority of the 125 samples investigated (56.8%, n = 71/125) (Table 3). Of these 18 types, GII.P16/GII.13 (n = 17; 100% of GII.13 capsid genotypes) was the most predominant inter-genotypic recombinant virus identified. GII.P7/GII.6 recombinant viruses were the second most predominant (n = 16; 94% of GII.6 capsid genotypes), followed by GII.Pb/GII.3 viruses (n = 11; 92% of GII.3 recombinants [GII.Pb, is now assigned GII.P21 by NoroNet]). Other recombinant viruses (n = 27) identified included; GII.P22/GII.5 (n = 8), GI.Pa/GI.3 (n = 3), GII.P16/GII.2, GII.Pg/GII.12 and GI.Pb/GI.6 (2 cases each), GII.P7/GII.5, GII.P12/GII.3, GII.P2/GII.7, GII.P16/GII.10, GII.Pb/GII.21, GII.P7/GII.9, GII.Pg/GII.1, GII.P16/GII.12, GII.P22/GII.6 and GI.Pf/GI.3 (1 case each) (Table 3).

**Table 3:** NoV wildtypes (non-GII.4) and intergenotypic recombinants identified in this study.

5' end ORF2^a^	Total ORF2 types identified	Subset analysed for recombination	3' end ORF1	Number (%) of ORF1 types identified	Location(s)
GI.1	1	1	Wildtype	1 (100)	NZ
GI.2	2	2	Wildtype	2 (100)	NZ
GI.3	26	15	Wildtype	11 (73.3)	NZ
			GI.Pa	3 (20)	NZ
			GI.Pf	1 (6.7)	NZ
GI.4	24	10	Wildtype	10 (100)	NZ
GI.5	2	2	Wildtype	2 (100)	NZ
GI.6	10	7	Wildtype	5 (71.4)	NZ
			GI.Pb	2 (28.6)	NZ
GI.7	2	1	Wildtype	1 (100)	NZ
GI.9	8	6	Wildtype	6 (100)	NZ
GII.1	1	1	GII.Pg	1 (100)	NSW
GII.2	22	5	Wildtype	3 (60)	WA
			GII.P16	2 (40)	NSW
GII.3	16	12	GII.Pb	11 (91.7)	All locations
			GII.P12	1 (8.3)	NZ
GII.5	11	9	GII.P22	8 (88.9)	All locations
			GII.P7	1 (11.1)	NZ
GII.6	28	17	GII.P7	16 (94.1)	All locations
			GII.P22	1 (5.9)	NZ
GII.7	32	12	Wildtype	11 (91.7)	All locations
			GII.P2	1 (8.3)	NZ
GII.9	1	1	GII.P7	1 (100)	NSW
GII.10	1	1	GII.P16	1 (100)	NZ
GII.12	3	3	GII.Pg	2 (66.7)	NZ
			GII.P16	1(33.3)	NZ
GII.13	28	17	GII.P16	17 (100)	All locations
GII.17	5	1	Wildtype	1 (100)	NSW
GII.20	1	1	Wildtype	1 (100)	WA
GII.21	1	1	GII.Pb	1 (100)	NSW
***Totals*:**	225	125	***Recombinants*:**	71	

^a^Excluded: GI.8 [n = 1], mixed (GI and GII) [n = 6], unknown GI [n = 1] and unknown GII [n = 6]

The remaining 54 GII NoV strains were identified as wild type viruses with identical genotypes for both polymerase and capsid sequences. These included GII.7 (n = 11/125, 8.8%), GII.2 (n = 3, 2.4%), GII.17 and GII.20 (n = 1, 0.8% each). The majority of GI viruses examined (70%, 38/54) were wild type viruses and included; GI.3 (20%), GI.4 (19%), GI.9 (11%), GI.6 (9%), GI.2 (4%), GI.5 (4%), GI.7 (2%) and GI.1 (2%) (Fig 4 and S2 Fig).

A subset of 25 GII.4 Sydney 2012 variants (n = 25) was also analysed for evidence of GII.4 intra-genotype recombination (S3 Fig). The majority of Sydney 2012 variants (n = 23, 85%) possessed the original GII.Pe ORF1. The remaining three strains were intra-genotypic recombinants with a Sydney 2012 ORF2/3 and a GII.P4 ORF1 that clustered with that of New Orleans 2009.

## Discussion

Despite improved awareness and infection control measures, NoV continues to cause significant morbidity and epidemics globally. This study investigated the molecular epidemiology and prevalence of NoV strains circulating in outbreaks and cases in two Australian states and in New Zealand between January 2013 and June 2014. The investigation was performed through the characterisation of the monthly occurrence of NoV-associated outbreaks and cases of acute gastroenteritis, as well as the distribution of circulating genotypes and recombinant viruses.

NoV has a distinct seasonality in temperate countries in the Northern hemisphere, which often results in significant increases in activity during winter months, or colder seasons of the year [45, 46]. The time frame of this study represented the period of early summer (from January) 2013, leading into the winter (June) of 2014 in the Southern hemisphere. In NSW, Australia, the incidence of NoV infection was relatively steady during the initial study period. However, during early spring, September and October 2013, a discernible increase of NoV positive cases and outbreaks were seen all across NSW (Fig 1A). This is consistent with our previous studies, which also reported a peak in NoV activity in NSW, Australia around September and October [21, 25]. In WA, Australia, the number of positive NoV samples and outbreaks were stable in early 2013, decreased in the winter of 2013, before rising sharply in late 2013 and remaining at elevated levels throughout the summer period (Fig 1B). The monthly trends in NoV outbreak numbers in New Zealand showed a similar pattern to WA, Australia, where increased NoV detections and outbreaks occurred during summer (March 2014). The peak outbreak activity in New Zealand during 2014 was delayed compared to previous seasons [25, 36], where NoV outbreaks peaked during spring every year from 2002 to 2012, except in 2011 [25]. To summarise over the study period, NSW experienced elevated NoV levels around October 2013 similar to previous years, whilst the following March (2014) increased NoV activity was seen in both WA and New Zealand.

A major epidemic of acute gastroenteritis in New Zealand in late 2012 was associated with GII.4 Sydney 2012 [25]. Interestingly, the number of reported NoV outbreaks in New Zealand was steady during 2013 without a clear epidemic peak (Fig 1B). This suggests that the magnitude of the previous season’s epidemic could reduce activity during the following season, as a consequence of more complete herd immunity. This is more likely if circulating viruses are unable to generate antigenic variation, as seen with the Sydney 2012 variant; which exhibited little variation at the known blockade epitopes during 2013 and 2014 (S1 Fig).

Unsurprisingly, genogroup II, particularly GII.4, was the major cause of NoV infection across all three locations examined in this study (Table 1, Fig 2). This is expected because, since 1996, variants of the NoV GII.4 lineage have caused 62 to 80% of all NoV cases globally [16, 47]. In Australia, NoV GII.4 was responsible for 74.8% and 70.1% of total NoV infections analysed in NSW and WA, respectively. While in New Zealand, GII.4 viruses were associated with 54.7% of total outbreaks identified during the same study period. In New Zealand, the proportion of GII.4 associated outbreaks (15.7%) was much lower between June and November 2013 (Fig 2C) compared to other months studied and interestingly coincided with overall lower NoV activity in the same period (Fig 1).

In the study period, there were more non-GII.4-associated outbreaks identified in New Zealand (45.2%) compared to both NSW and WA in Australia (25.2 and 29.9%, respectively) (Table 1). This may be partly explained by a higher proportion of catered/commercial food settings, childcare centres or other community acquired disease reported in New Zealand compared to Australia, because GI viruses are associated more with foodborne transmission compared to GII viruses [48]. In addition NoV outbreaks in childcare centres were relatively common in New Zealand (11% of outbreaks) (Table 2), and non-GII.4 strains including GII.Pb/GII.3, GII.2, GII.6, GII.7 and GII.13 have shown a predominance to infect children [49–53], which again could account for some of the genogroup and genotype differences seen between viruses from Australia and New Zealand.

In many countries the reported settings for the NoV outbreaks varies; however, they are most predominant within institutional settings such as hospitals and aged-care facilities [54]. Between 1995–2000, most reported NoV outbreaks in England and Wales (78%), Spain (64%) and The Netherlands (55%), occurred in hospitals and residential homes [55, 56]. The present study was consistent with these finding and demonstrated that aged-care facilities were the predominant settings for NoV outbreaks reported in both Australia and New Zealand (Table 2). This is also similar to the US, where the majority of outbreaks (60%) between 2009 to 2013 were also reported in long-term care facilities [57].

Recombination is a major driving force of viral evolution, enabling viral genomic diversification and thus increasing selective advantages. For NoV, recombination is most commonly identified at the ORF1-ORF2 junction [23, 42], therefore, we screened a selection (n = 125) of representative GI and GII strains from this study to identify potential recombinants at this break point. Inter-genotype recombination, particularly in NoV GII, was commonly detected in this cohort, with 15 out of 18 different recombinant types detected belonging to GII NoV (92%, n = 65/71) (Table 3). Of these GII recombinants, GII.P16/GII.13, GII.P6/GII.7, GII.Pb/GII.3 and GII.P22/GII.5 recombinants were identified across all geographical locations (Table 3, Fig 3 and S3 Fig). The distribution of these genotypes from all regions in phylogenetic trees also suggests they may have been introduced through human travel. The three remaining recombinant types were all GI recombinants and isolated from NoV outbreaks that occurred in New Zealand (Fig 4 and S2 Fig). These recombinant strains have acquired orphan polymerases [13], pORF1 (GI.Pa, GI.Pb, and GI.Pf), as assigned by NoroNet [24], whilst the ORF2 was one of the commonly identified GI.3 or GI.6. These GI recombinant types have also been detected in Japan and China (GenBank accession numbers: GQ856473, GQ856464 and AB187514) [58].

Previous studies have revealed that the contemporary GII.4 variants, New Orleans 2009 and Sydney 2012 have undergone a complex evolution process involving intra-genotype recombination at the ORF1/ORF2 overlap [23]. In the present study, a subset of GII.4 Sydney 2012 strains (n = 25) were examined for GII.4 intra-genotypic recombination (S3 Fig). Three Sydney 2012 variants from New Zealand were shown to be GII.4 New Orleans 2009 (ORF1)—Sydney 2012 (ORF2) recombinants. The recombination between the two GII.4 pandemic variants has also been identified in Italy [59]. The remaining 22 Sydney 2012 strains studied here possessed the signature GII.Pe ORF1 as reported previously [25]. This suggests that the GII.Pe/GII.4 Sydney 2012 was the most dominant variant.

Reports in 2015 have identified a potential new pandemic NoV, termed Kawasaki GII.17 [60–62]. Of note, a GII.P17/GII.17 strain (GenBank accession KT239640B) identified from this study shared 99% nt identity across the 575 nt ORF1/ORF2 overlapping region compared to this emerging Kawasaki GII.17 strain (AB983218) [60]. The strain was isolated from a sporadic case of acute gastroenteritis in NSW during May 2014 (Fig 3 and S3 Fig). In WA, three GII.P17/GII.17 strains were reported from sporadic cases between February and April 2014 and it was also identified from a New Zealand outbreak in April 2014. The GII.17 polymerase was initially identified as novel [63]; however has recently been classified as GII.P17 by NoroNet. The identification of this strain in the Oceania region is important as it confirms this emergent virus is increasing in prevalence globally and could be associated with rising NoV activity in Australia and New Zealand during 2016.

## Conclusions

In conclusion, the GII.4 lineage continues to cause major NoV epidemics in Australia and New Zealand. Following its epidemic emergence in 2012, up until at least June 2014, the GII.4 variant Sydney 2012 was still the predominant NoV strain in circulation in Australia and New Zealand. The widespread genetic diversity of circulating strains was observed in 239 non-GII.4 strains. Among these at least 18 different NoV recombinant types were identified, with the majority belonging to GII viruses. In light of this, continuous and thorough surveillance of NoV-associated gastroenteritis will be essential to further understand how novel variants emerge. This research will inform the composition of vaccine candidates that are currently in development. Based on the known NoV diversity, frequent recombination and their propensity to generate antigenic variation, the composition of any NoV vaccine will likely need to be updated based on complete and timely molecular epidemiology studies.

## Supporting Information

S1 Fig Antigenic variation in the GII.4 Sydney 2012 capsid sequence. Residue positions within the capsid are shown above the amino acid residues. Labelled boxes above each position indicate sites within known blockade epitopes A-E, that are important determinants of viral antigenicity. Amino acids have been coloured based on the properties of their side-chains: blue for positive charged—R and H; red for negative charged—D; green for polar uncharged—S and N; yellow for hydrophobic—A, V, I, L, M; pink for special cases—P and G.
(TIF)

S2 Fig Phylogenetic analysis of NoV GI partial polymerase (Region B) sequences from New Zealand. Neighbour-Joining phylogeny of 172-bp sequences from GI viruses were generated using programs in MEGA 5. Representative NoV sequences determined in this study (n = 43) are coloured in red. Reference sequences (n = 14) were obtained from GenBank and labelled with accession numbers in black (see S1 Table for strain details). Bootstrap percentage values are shown at each branch point for values ≥75% (1000 replicates). The distance scale represents the number of nucleotide substitutes per site.
(PDF)

S3 Fig Phylogenetic analysis of NoV GII partial polymerase (Region B) sequences. Representative NoV sequences determined in this study (n = 107) are coloured, with blue, green and red representing samples collected from NSW Australia, WA Australia and New Zealand, respectively. The sequences identified in this study are labelled by Sample ID/Collection month and year/Country. Bootstrap percentage values are shown at each branch point for values ≥75% (1000 replicates). The distance scale represents the number of nucleotide substitutes per site.
(PDF)

S1 Table Reference noroviruses used to construct phylogenetic trees in this study. (PDF)
