# Supplementary figures and images for: A Multi-Site Study of Norovirus Molecular Epidemiology in Australia and New Zealand, 2013-2014
Source: PLoS One. 2016 Apr 26;11(4):e0145254. doi: 10.1371/journal.pone.0145254 (PMC4846056; doi:10.1371/journal.pone.0145254)

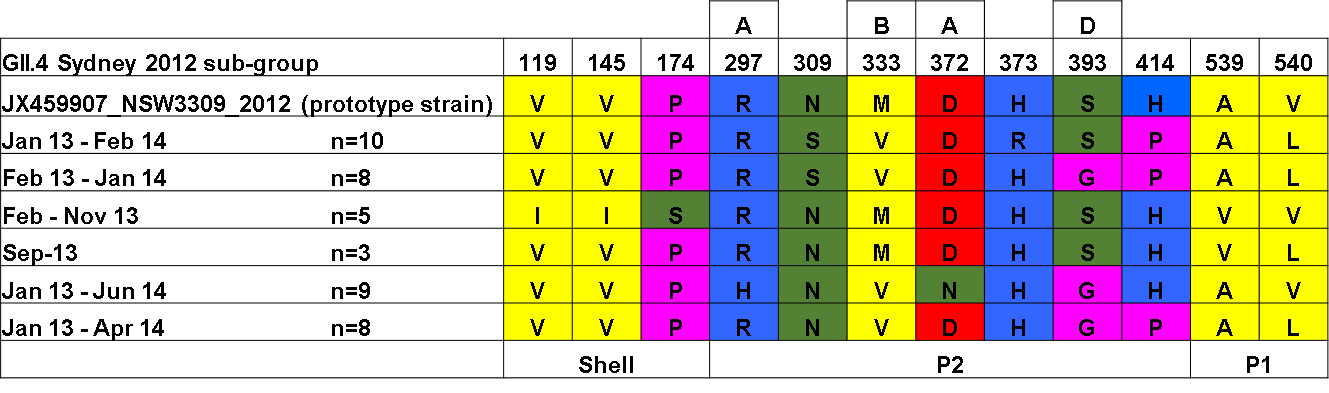

Supplement: S1 Fig — Residue positions within the capsid are shown above the amino acid residues. Labelled boxes above each position indicate sites within known blockade epitopes A-E, that are important determinants of viral antigenicity. Amino acids have been coloured based on the properties of their side-chains: blue for positive charged—R and H; red for negative charged—D; green for polar uncharged—S and N; yellow for hydrophobic—A, V, I, L, M; pink for special cases—P and G. (TIF) [file pone.0145254.s001.tif]

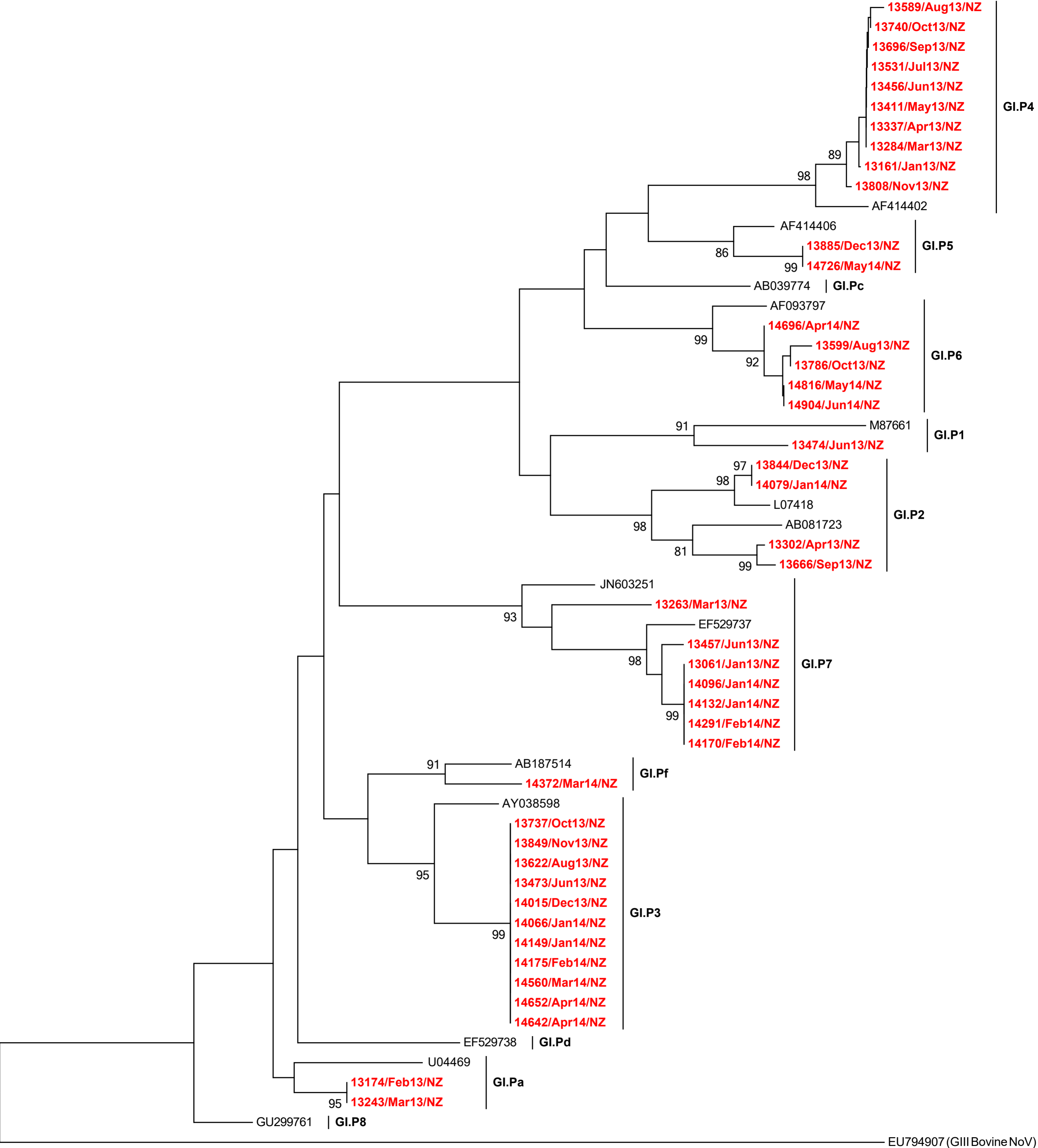

0.1

Supplement: S2 Fig — Neighbour-Joining phylogeny of 172-bp sequences from GI viruses were generated using programs in MEGA 5. Representative NoV sequences determined in this study (n = 43) are coloured in red. Reference sequences (n = 14) were obtained from GenBank and labelled with accession numbers in black (see S1 Table for strain details). Bootstrap percentage values are shown at each branch point for values ≥75% (1000 replicates). The distance scale represents the number of nucleotide substitutes per site. (PDF) [file pone.0145254.s002.pdf]

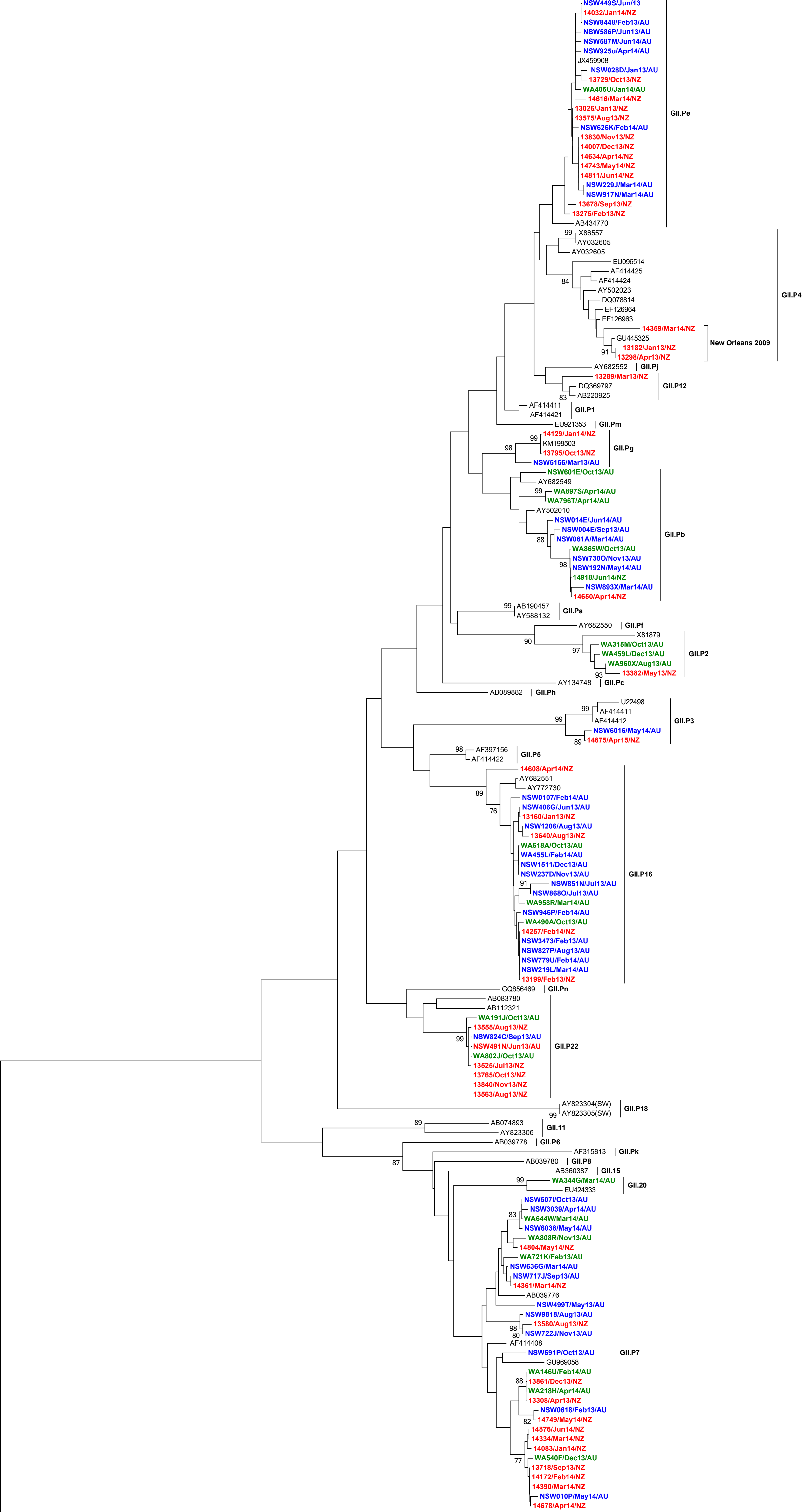

0.1

EU794907 (GIII Bovine NoV)

Supplement: S3 Fig — Representative NoV sequences determined in this study (n = 107) are coloured, with blue, green and red representing samples collected from NSW Australia, WA Australia and New Zealand, respectively. The sequences identified in this study are labelled by Sample ID/Collection month and year/Country. Bootstrap percentage values are shown at each branch point for values ≥75% (1000 replicates). The distance scale represents the number of nucleotide substitutes per site. (PDF) [file pone.0145254.s003.pdf]
