# Supplementary material for: A Multi-Site Study of Norovirus Molecular Epidemiology in Australia and New Zealand, 2013-2014
Source: PLoS One. 2016 Apr 26;11(4):e0145254. doi: 10.1371/journal.pone.0145254 (PMC4846056; doi:10.1371/journal.pone.0145254)
Supplement: S1 Table — (PDF) [file pone.0145254.s004.pdf]

S1 Table. Reference NoVs used to construct phylogenetic trees in this study.

GenBank Accession	Reference NoV (Host/genotype/strain identifier/collection year/country of origin)
KF429765	Hu/GI.1/8W/1968/US
KF306212	Hu/GI.2/Jingzhou2013401/2013/CHN
AF145709	Hu/Stav/1995/NOR
EF547396	Hu/GI.3/Akabane991130-2258/1999/JPN
JN603244	Hu/GI.3/Lilla Edet S29/2008/SWE
AB042808	Hu/GI.4/Chiba407/1987/JPN
JN603273	Hu/GI.4/Lila Edet S45/2008/SWE
AJ277614	Hu/GI.5/Musgrove/1989/UK
JQ388274	Hu/GI.6/Kingston ACT160D/2010/AUS
AJ277615	Hu/GI.6/Sindlesham/1995/UK
JN603265	Hu/GI.7/Lilla Edet S23/2008/SWE
AJ277609	Hu/GI.7/Winchester/1994/UK
AJ844469	Hu/GI.7/Chiba 030100/2003/JPN
AF538679	Hu/GI.8/Boxer/2001/USA
GU296356	Hu/GI.9/Lilla Edet p2/2008/SWE
HQ637267	Hu/GI.9/Vancouver730/2004/CAN
M87661	Hu/GI.1/Norwalk/1993/USA
L07418	Hu/GI.2/Southampton/1993/UK
AY038598	Hu/GI.P3/VA98115/1998/USA
AF414402	Hu/GI.P4/New Orleans 66/1993/US
AF414406	Hu/GI.5/Appalachicola Bay 318/1995/US
AF093797	Hu/GI.6/Norwalk/1997/DE
JN603251	Hu/GI.7/Lila Edet S5b/2008/SWE
GU299761	Hu/GI.8/2008890321/2008/US
EF529737	Hu/GI.P9/Chatellerault709/2004/FR
U04469	Hu/GI.P9-GI.3/Desertm Shield DSV395/1993/US
AB081723	Hu/GI.Pb/WUG1/2002/JPN
AB039774	Hu/GI.Pc-GI.5/SzUG1/2002/JPN
EF529738	Hu/GI.Pd/Vesoul576/2003/FR
AB187514	Hu/GI.Pf-GI.3/Otofuke/1979/JPN
AY580335	Hu/GII.1/Courdon78/2000/FR
AY919139	Hu/GII.1/Picton/2003/AU
U07611	Hu/GII.1/Hawaii/1971/US
AF414421	Hu/GII.P1-GII.1/Port Canaveral 301/1994/US
AF414411	Hu/GII.P1-GII.3/Lionville 247/1993/US
DQ366347	Hu/GII.2/OsakaNI/2004/JPN
X81879	Hu/GII.P2-GII.2/Melksham/UK
AB242258	Hu/GII.3/78Ru/2004/JPN
GU980585	Hu/GII.3/CBNU1/2006/KOR
U02030	Hu/GII.3/TV24/1993/US

GenBank Accession	Reference NoV (Host/genotype/strain identifier/collection year/country of origin)
AF414412	Hu/GII.P3/New Orleans 279/1994/US
U22498	Hu/GII.P3-GII.3/MX/US
AB303932	Hu/GII.4/DenHaag001/2003/NL
AB445395	Hu/GII.4/Apeldoorn317/2007/NL
EU876890	Hu/GII.4/Dijon-E1057/2002/FR
FJ514242	Hu/GII-4/CUK-3/2008/KR
GQ845367	Hu/GII-4/Orange/NSW001P/2008/AU
HQ009513	Hu/GII.4/JB-15/2008/KR
JQ613517	Hu/GII.4/WA210Z/2007/AU
JX629458	Hu/GII.4/Hong Kong CUHK3655/2012/CH
KC175323	Hu/GII.4/Hong Kong CUHK3630/2012/CH
AF414424	Hu/GII.4/MiamiBeach326/1995/US
AF414425	Hu/GII.4/Burwash Landing 331/1995/US
AY032605	Hu/GII.4/MD145-12/1987/US
AY502023	Hu/GII.4/Farmington Hills/2002/US
DQ078814	Hu/GII.4/Hunter504D 04O/2004/AU
EF126963	Hu/GII.4/Yerseke38/2006/NL
EF126964	Hu/GII.4/Terneuzen70/2006/NL
EU096514	Hu/GII.4/Kapuvar3029/2007/HUN
GU445325	Hu/GII.4/New Orleans1805/2009/US
JX459908	Hu/GII.4/Sydney/NSW514/2012/AU
X76716	Hu/GII.4/Bristol/1993/UK
X86557	Hu/GII.4/Lordsdale/1995/UK
AB212306	Hu/GII.5/Hokkaido 133/2003/JPN
AF414423	Hu/GII.5/White River/290/1994/US
AJ277607	Hu/GII.5/Hillingdon/90/UK
AF397156	Hu/GII.P5-GII.5/MOH/1999/US
AF414422	Hu/GII.P5-GII.5/New Orleans 306/1994/US
AF414410	Hu/GII.6/Miami/292/1994/US
AJ277620	Hu/GII.6/Seacroft/1990/UK
HM633213	Hu/GII.6/Shizuoka 8913/2008/JPN
AB039778	Hu/GII.P6-GII.6/Saitama U16/JPN
AF414409	Hu/GII.7/Gwynedd 273/1994/US
AJ277608	Hu/GII.7/Leeds/1990/UK
AB039776	Hu/GII.P7-GII.6/Saitama U3/JPN
AF414408	Hu/GII.P7-GII.6/Maltimore 274/1993/US
AF195848	Hu/GII.8/Amsterdam 98-18/1998/NL
AB039780	Hu/GII.P8-GII.8/Saitama U25/JPN
AY038599	Hu/GII.9/VA97207/1997/US
AF427118	Hu/GII.10/Erfurt 546/2000/DE
AY237415	Hu/GII.10/Mc37/2003/JPN
AB074893	Sw/GII.11/Sw918/1997/JP

GenBank Accession	**Reference NoV (Host/genotype/strain identifier/collection year/country of origin)**
AY823306	Sw/GII.P11-GII.19/OH-QA170/2003/US
AB039775	Hu/GII.12/Saitama U1/2002/JPN
AB044366	Hu/GII.12/Hiroshima 9912-02F/1999/JPN
AJ277618	Hu/GII.12/Wortley/90/UK
AB220925	Hu/GII.P12-GII.4/Chiba/04-974/2004/JP
DQ369797	Hu/GII.P12-GII.4/Guangzhou NVgz01/CH
GU969058	Hu/GII.13/Maizuru 8679/2008/JPN
AY113106	Hu/GII.13/Fayetteville/1998/US
AY682548	Hu/GII.13/Pont de Roide 671/2004/FR
AY130761	Hu/GII.14/M7/1999/US
AY130762	Hu/GII.15/J23/1999/US
AB360387	Hu/GII.P15/Hiroshima 66-1110/2006/JPN
AY772730	Hu/GII.P16/Neustrelitz260/2000/DE
AY682551	Hu/GII.P16-GII.16/VannesL23/1999/FR
AB983218	Hu/GII.P17-GII.17/Kawasaki323/2014/JPN
AY502009	Hu/GII.17/CS-E1/2002/US
AY823305	Sw/GII.P18/OH-QW125/2003/US
AY823304	Sw/GII.P18/GII.18/OH-QW101/2003/US
EU424333	Hu/GII.P20/Leverkusen267/2005/DE
AB542917	Hu/GII.20/OC07118/2007/JPN
EU373815	Hu/GII.20/Luckenwalde591/2002/DE
AY675554	Hu/GII.21/IF1998/2003/Iraq
EU019230	Hu/GII.21/Ahm PC03/2006/IND
AY682549	Hu/GII.P21/Pont de Roide 673/2004/FR
AY502010	Hu/GII.P21-GII.16/Tiffin/1999/US
AB112321	Hu/GII.P22/Saitama T66eGII/02/JPN
AB083780	Hu/GII.P21-GII.21/Yuri/JPN
AB190457	Hu/GII.Pa/SN2000JA/JPN
AY588132	Hu/GII.Pa/Sydney 2212/2003/AU
AY134748	Hu/GII.Pc-GII.2/Snow Mountain/US
AB434770	Hu/GII.Pe-GII.4/OC07138/2007/JPN
AY682550	Hu/GII.Pf-GII.5/S63/1999/FR
KM198503	Hu/GII.Pg/C2033/2010/UK
GQ845370	Hu/GII.g-GII.12/StGeorge/NSW199U/2008/AU
AB089882	Hu/GII.Ph/OC97007/1997/JPN
AY682552	Hu/GII.Pj-GII.2/Crete E3/1997/FR
AF315813	Hu/GII.Pk/OC96065/1996/JPN
EU921353	Hu/GII.Pm/Pune PC24/2006/IND
GQ856469	Hu/GII.Pn/Beijing853931/2007/CH
EU794907	Bo/GIII/B309/2003/BEL

Host: Hu – Human; Sw – Swine; Bo – Bovine

Country: US – BEL – Belgium; CAN – Canada; CHN – China; DE – Germany; FR – France; HUN – Hungary; IND – India; JPN – Japan; KOR – Korea; NL – Netherlands; NOR – Norway; SWE – Sweden; UK – United of Kingdoms and US – Unite of Sates;
